# Analysis of spatial-temporal distribution characteristics and natural infection status of SFTS cases in Hefei from 2015 to 2021

**DOI:** 10.1265/ehpm.23-00149

**Published:** 2023-11-15

**Authors:** Qi Zhang, Wenwen Liu, Wenjing Wang, Linlin Zhang, Juan Li, Renshu Tang, Jing Jin, Wei Chen, Lei Zhang

**Affiliations:** Hefei Center for Disease Control and Prevention, Hefei, China

**Keywords:** SFTS, Infection status, Spatial-temporal distribution

## Abstract

**Background:**

To analyze the prevalence and spatial-temporal characteristics of severe fever with thrombocytopenia syndrome (SFTS), clustering mode of transmission, and the serological dynamic detection results in multiple areas in Hefei from 2015 to 2021, and to provide the basis for SFTS prevention and control.

**Method:**

Case data were obtained from the Chinese Disease Control and Prevention Information System. Information on the clustering outbreak was obtained from the outbreak investigation and disposal report. Population latent infection rate information was obtained from field sampling in multiple-incidence counties in 2016 and 2021 by multi-stage random sampling. Epi data3.2 and SPSS 16.0 softwares were used to perform a statistical analysis of the data on SFTS cases, and QGIS 3.26 software was used to draw the incidence map with township (street) as unit.

**Results:**

The an average annual reported incidence rate of SFTS in Hefei from 2015 to 2021 was 0.65/100,000, and the case fatality rate was 9.73%. The overall prevalence of SFTS epidemics in Hefei City showed a fluctuating upward trend from 2015 to 2021 (χ^2^trends = 103.353, *P* < 0.001). Chaohu City, Feixi County, Feidong County and Lujiang County ranked the top 4 in the city in terms of average annual incidence rate. The number of epidemic-involved towns (streets) kept increasing ((χ^2^_trend_ = 47.640, P = 0.000)). Co-exposure to ticks accounted for the majority of clustered outbreaks and also human-to-human outbreaks. Population-based latent infection rate surveys were conducted in four SFTS multi-incidence counties, with 385 people surveyed in 2016 and 403 people surveyed in 2021, increasing the population-based latent infection rate from 6.75% to 10.91%, just as the incidence rate increased.

**Conclusions:**

The incidence rate of SFTS in Hefei is obviously regional, with an expanding trend in the extent of the epidemic involved. Co-exposure to ticks accounted for the majority of clustered outbreaks and the latent infection rate cannot be ignored.

From late March to mid-July 2009, an new infectious disease identified as severe fever with thrombocytopenia syndrome (SFTS) was reported in rural areas of Hubei and Henan provinces in central China, with SFTS virus (SFTSV) as the pathogen [1, 2]. SFTSV is generally spherical, 80∼100 nm in diameter, and its genome contains three single negative stranded RNA fragments (L, M and S) [3]. SFTSV is sensitive to heat and can be completely inactivated at 60 °C for 30 min. SFTSV is not acid-resistant, sensitive to ultraviolet light, ether, chloroform and formaldehyde, and also sensitive to common chlorine-containing disinfectants such as hypochlorous acid [1]. The World Health Organization classified SFTS as one of the most serious infectious diseases in 2017 [4].

Currently, the main endemic area for SFTS is central China, and SFTVS infection has been reported in Japan, Korea, Vietnam, and the United States [1, 5–8]. According to the Chinese Disease Prevention and Control Information System, the overall incidence of SFTS in China increased from 2011–2021, with an average annual percentage change in incidence of 14.80% [9]. Incidence was also highly spatially clustering, with 99.23% of cases concentrated in seven provinces, including Anhui, Henan, Shandong, Hubei, Jiangsu, Zhejiang, and Liaoning, and 70.28% were concentrated in 11 prefecture-level cities in these seven provinces [9]. The geographical distribution showed a trend of spreading from central to northeast, west and south [9].

Studies have shown that SFTS, as a tick-borne zoonosis, is transmitted mainly through tick biting and blood sucking, that is, people engaged in outdoor or agricultural activities will have become latent infection or even develop the SFTS after being bitten by ticks carrying SFTSV [10]; Secondly, human-to-human transmission is achieved in healthy people through contact with body fluids or excreta of SFTS, a situation that is common among family members caring for patients and medical personnel involved in treatment and nursing [11, 12]. This is common among family members caring for patients and medical personnel involved in life-saving care [11, 12]. Anhui province is an endemic area for SFTSV, with several outbreaks of interpersonal transmission [13].

Hefei now has jurisdiction over four counties: Feidong, Feixi, Changfeng and Lujiang, one county is Chaohu City, and four districts: Yaohai, Luyang, Shushan and Baohe [14]. The territory of Hefei is mainly hilly and granite, and the Jianghuai watershed crosses the whole territory from west to east [15]. The terrain of the main urban area is inclined from northwest to southeast, with undulating hills; the southwest part of the Dabie mountain afterpulse, with mountain ranges [15]. Hefei is located in the mid-latitude area, a subtropical monsoon humid climate, monsoon obvious, four distinct seasons, mild climate, moderate rainfall [15].

There are asymptomatic infected individuals in SFTS [16], and SFTSV has a high seropositivite rate in healthy populations in central and eastern China. However, the season, the sample size and other reasons of the study led to heterogeneous results. In contrast, about 8.3% of SFTS were missed in highly endemic areas, and the actual incidence of SFTS was higher than the currently reported level [17]. Therefore, we used the same sampling and testing methods to carry out two consecutive surveys of hidden infection rate, which would help to obtain a more realistic latent infection rate and its changing trend, and to take corresponding preventive and control measures.

By analyzing the trends of spatial-temporal characteristics, the transmission mode of clustering outbreaks, and the results of serology dynamic testing of SFTS in Hefei from 2015 to 2021, this study explored the possible influencing factors as well as the key areas and populations requiring attention in the future, so as to take more targeted measures and provide a basis for scientific epidemic prevention and control.

## 1 Materials and methods

### 1.1 Diagnostic criteria

According to the Guidelines for Prevention and Treatment of Fever with Thrombocytopenia Syndrome (2010) of the former Ministry of Health [18]: (1) Suspected cases: patients with epidemiological history (history of working, living or traveling in hilly, forest, and mountainous areas during the epidemic season, or history of tick bite within 2 weeks before the onset of the disease), fever and other clinical manifestations, and decreased platelet and white blood cells in peripheral blood; (2) Confirmed cases: Suspected cases have one of the following: Case specimens tested positive for novel Bunyavirus nucleic acid; The titer of new Bunyavirus IgG antibody in positive or recovery period was more than 4 times higher than that in acute period. A novel Bunyavirus was isolated from case specimens. (3) Cluster cases: Within 2 weeks, there are 2 or more cases in the same village, or in the same hillside, forest, tea garden, scenic spot, etc., among people working or traveling, or similar cases in close contacts of the case.

### 1.2 Data source

Information on confirmed cases was obtained from the “SFTS surveillance database” for nucleic acid-positive cases with an onset address in Hefei City between January 1, 2015 and December 31, 2021. Information on the clustering outbreak was obtained from the outbreak investigation and disposal report. Population latent infection rate information was obtained from field sampling in multiple-incidence counties in 2016 and 2021. The number of townships (streets) was obtained from the annual statistical yearbook of Hefei, and the population data were obtained from the “Infectious Disease Reporting Information Management System”.

### 1.3 Survey methodology

#### 1.3.1 Case investigation

Epidemiological case investigation was conducted and tracking laboratory test results after obtaining case information through the daily browsing of the “Infectious Disease Reporting Information Management System”. The Center for Disease Control and Prevention (CDC) in the place of case onset receives the epidemiological investigation data after the first review and conducts additional investigation and prognosis referral follow-up, which is finally reviewed by Hefei CDC and then archived and entered into the “SFTS surveillance database”.

#### 1.3.2 Population latent infection rate survey

The multi-stage random sampling method was used in both 2016 and 2021 to select the top 4 multi-incidence counties reporting the number of confirmed SFTS cases among the 9 counties under the jurisdiction of Hefei, and then select one township each, and then randomly select a natural village in the selected township as the investigation site. According to the peak incidence rate of confirmed SFTS cases [19], June was determined as the investigation time. Then recruit 95–100 healthy people from the selected natural village for questionnaire survey and blood specimen collection.

Healthy individuals were defined as those who had lived in the selected natural village for more than 1 year, were 3 years of age and older, and had no history of fever or other discomfort in the 2 weeks prior to sampling. People who had been diagnosed with SFTS in the past were also excluded from the study.

### 1.4 Data processing methods

#### 1.4.1 Data processing and statistics

Epi data3.2 was used to establish the case and population infection survey databases respectively, entered into the database, exported to Excel and then organized and plotted the corresponding graphs. The chi-square test was performed using SPSS 16.0. α = 0.05 was the test level.

#### 1.4.2 Spatial thematic map production

The QGIS 3.26 software was applied to vectorize the geographic boundaries of the administrative divisions of townships (streets) in Hefei, and to map the spatial distribution of the townships (streets) with confirmed SFTS cases by year. The number of cases was divided into four classes in the order of 0 (no cases), 1–5 cases, 6–10 cases, 11–15 cases and 15–20 cases by township (street).

## 2 Results

### 2.1 Overall epidemic trend

**Fig. 1:**
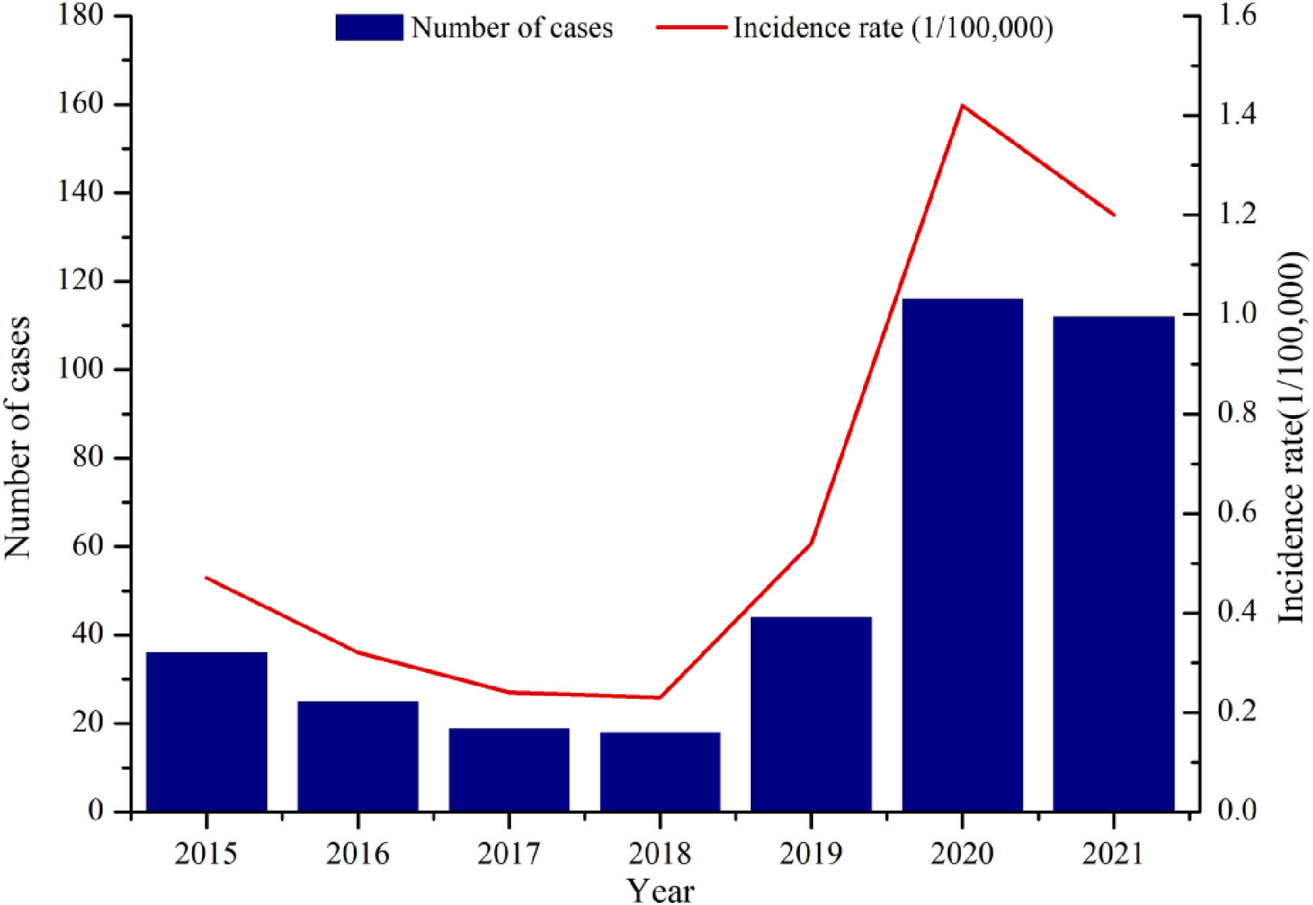
The overall epidemic trends of SFTS in Hefei, 2015–2021

It’s reported 370 confirmed SFTS cases from 2015 to 2021 in Hefei, with an average annual reported incidence rate of 0.65/100,000. There were 36 cases of death, and the case fatality rate was 9.73%. Of which, the incidence rate decreased year by year during 2015 to 2018, with the lowest in 2018 (0.23/100,000) and rebounded rapidly after 2019, with the incidence rate in 2020 and 2021 at the highest level during the 7-year period, reaching 1.42/100,000 and 1.20/100,000, respectively. The overall trend was upward (χ^2^trends = 103.353, *P* < 0.001). (see Fig. 1).

### 2.2 County-level distribution

**Fig. 2:**
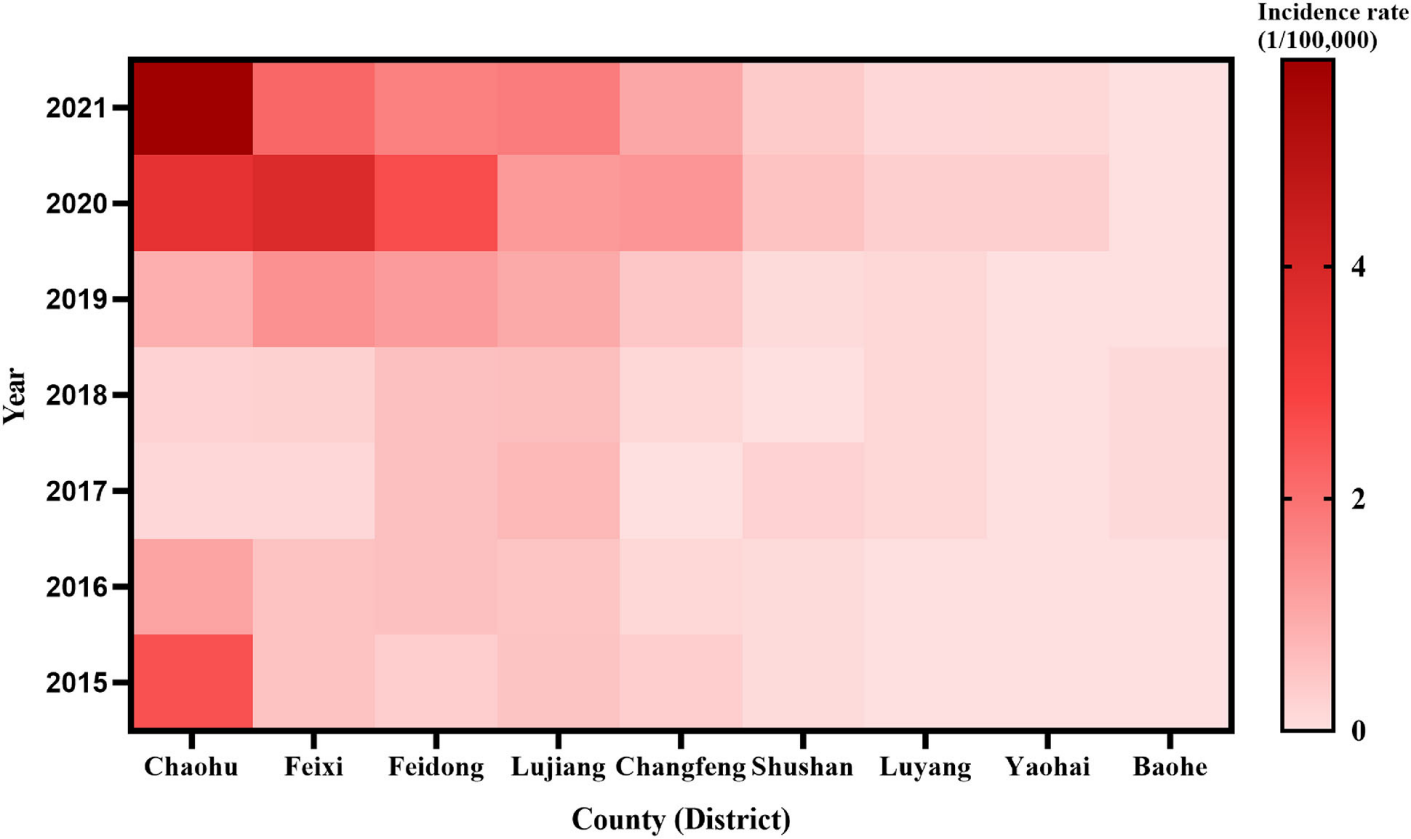
Incidence trends in counties (districts) with confirmed SFTS cases in Hefei, 2015–2021

There were cases in all 9 counties (districts) under the jurisdiction, but the incidence rate in the surrounding counties was higher than that in the main urban area. The average annual incidence rate is higher than Hefei average annual incidence rate (0.65/100,000) in Chaohu City (1.98/100,000), Feixi County (1.31/100,000), Feidong County (1.09/100,000) and Lujiang County (0.89/100,000). The number of cases in the 4 counties ranked the top four in Hefei, accounting for 84.59% of the total cases. The incidence rates of the other five counties were lower than the city’s average annual incidence level (see Fig. 2).

From each year, the incidence rate of Shushan District and Luyang District exceeded that of Chaohu City and Feixi County in 2017, ranking in the top four; the incidence rate of Changfeng County reached 1.33/100,000 in 2020, slightly higher than that of Lujiang County, entering the top 4; the counties with the top 4 incidence rates in the remaining years have been Chaohu City, Feixi County, Feidong County and Lujiang County, but with slight changes in the order (see Fig. 2).

### 2.3 Township-level distribution

#### 2.3.1 Prevalence range

**Fig. 3:**
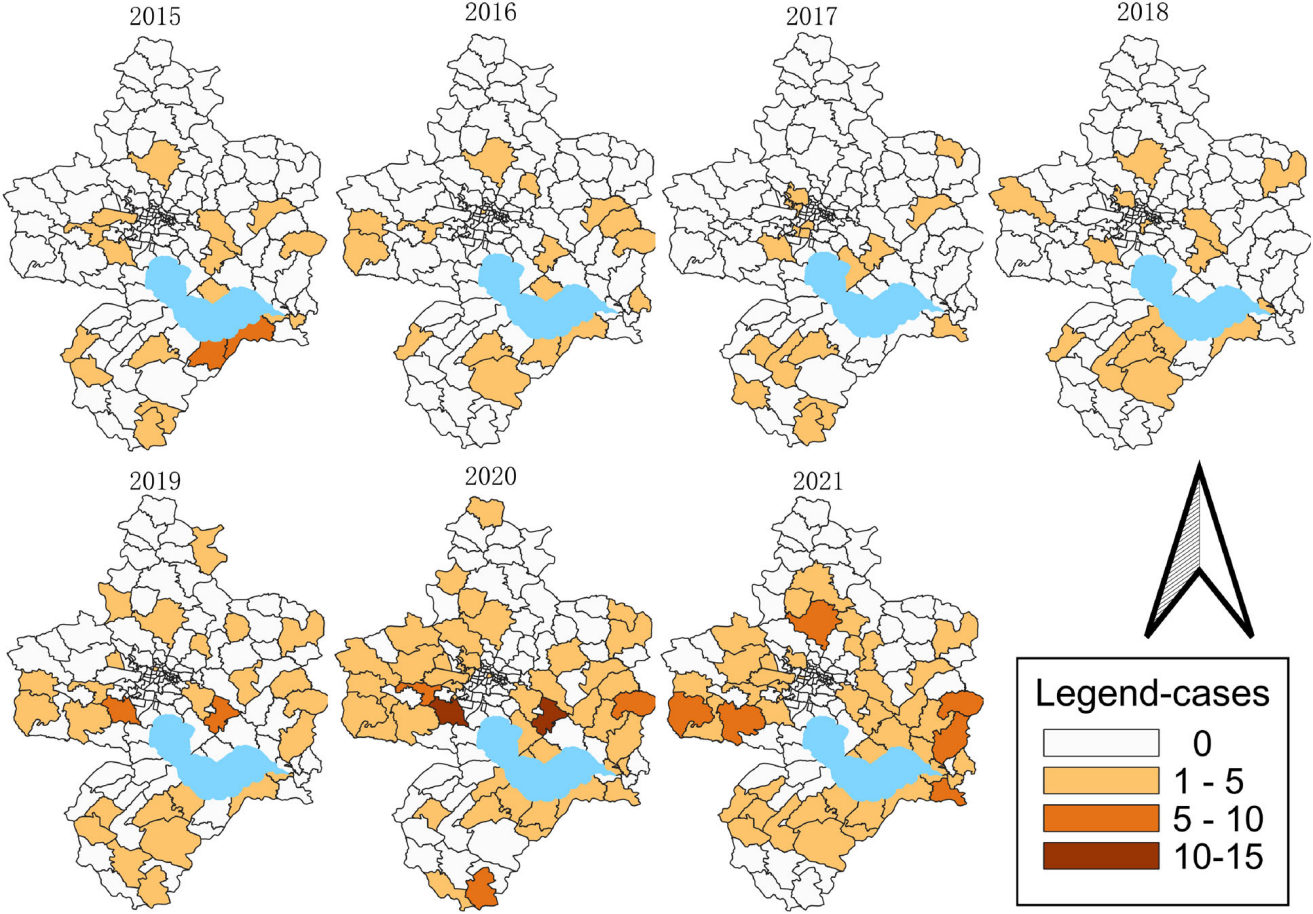
Spatial-temporal dynamic distribution of townships (streets) with confirmed SFTS cases in Hefei, 2015–2021

The area involved in SFTS cases in Hefei is expanding, and there is a trend of covering the townships (streets) in the county and spreading to the main urban area. 7 years, the composition ratio of the number of townships (streets) with confirmed SFTS cases to the total number of townships (streets) in that year ranged from 12.50% to 38.89%, and the composition ratios from 2015 to 2021 were 14.06% (18/128), 13.28% (17/128), 12.50% (16/128), 12.80% (16/125), 22.22% (28/126), 38.10% (48/126) and 38.89% (49/126) respectively. The difference in the composition ratio of the epidemic-involved towns (streets) was statistically significant among years (χ^2^ = 63.915, *P* < 0.001), and the number of epidemic-involved towns (streets) kept increasing (χ^2^_trend_ = 47.640, *P* < 0.001) (see Fig. 3).

#### 2.3.2 Epidemic intensity

The incidence pattern of SFTS in Hefei coexisted with a highly sporadic and locally cluster. The cumulative number of cases in the townships (streets) ranged from 1 to 28, with a median of 3. The number of townships (streets) with 1–5 cumulative cases accounted for 42.03% of the total cumulative townships (streets) during the 7-year period, 14.29% for 6–10 cases, 8.06% for 11–15 cases, 5.66% for 16–20 cases, and 3.70% for 21 cases and above.

From 2015 to 2021, the number of towns (streets) with 1–5 cases was relatively low in the first four years, and then increased year by year, with a composition ratio of 12.50% to 36.51%. The composition ratio of towns (streets) with 6–10 cases increased from 0% to 0.79% in 2015–2019 to 2.38% in 2020–2021. 2 towns (streets) had 11–15 cases of SFTS (see Table 1).

**Table 1:** Epidemic intensity of confirmed SFTS in Hefei, 2015–2021 [n/%]

**The number of cases**	**2015**	**2016**	**2017**	**2018**	**2019**	**2020**	**2021**
0	110/85.94%	111/86.72%	112/87.50%	109/87.20%	98/77.78%	78/61.90%	77/61.11%
1–5	17/13.28%	17/13.28%	16/12.50%	16/12.80%	27/21.43%	43/34.13%	46/36.51%
6–10	1/0.78%	0	0	0	1/0.79%	3/2.38%	3/2.38%
11–15	0	0	0	0	0	2/1.59%	0

### 2.4 Clustering outbreaks

Eight SFTS clustering outbreaks occurred in 7 years, involving 7 towns (streets) in 4 counties. 21 cases were reported in the 8 outbreaks, with 7 deaths and a 33.33% case fatality rate. Follow-up investigation of 67 close contacts, 4 cases of latent infection were detected, with the latent infection rate of 5.97%.

Five of the eight outbreaks (62.50% of the number of outbreaks) were transmitted by co-exposure to ticks and three (37.50% of the total number of outbreaks) by human-to-human transmission. The difference between the two modes of transmission leading to the case fatality rate was not statistically significant by Fisher’s exact probability test (P = 1.000) and the difference in the latent infection rate was not statistically significant (P = 0.127). There was no statistically significant difference between the two different discovery pathways leading to the case fatality rate (P = 0.624) (see Table 2).

**Table 2:** Basic information of SFTS clustering outbreaks, 2015–2021

**Year**	**Area**	**Number of cases involving epidemic diseases**	**Number of case fatality**	**Close contact disposal situation**		**Possible ** **transmission ** **method**	**Discovery ** **pathway**

				**Number of ** **Sampling**	**Number of ** **latent infection**		
2016	Sanbing Town, Chaohu City	2	1	12	4	Human-to-human	Passive surveillance
2018	Qiaoji Town, Feidong County	3	1	3	0	Human-to-human	Passive surveillance
2019	Qiaoji Town, Feidong County	2	2	5	0	Co-exposure to ticks	Passive surveillance
2020	Bazhen Town, Chaohu City	2	0	6	0	Co-exposure to ticks	Passive surveillance
2020	Changlin Town, Feidong County	2	1	4	0	Co-exposure to ticks	Passive surveillance
2020	Alum Mountain Town, Lujiang County	3	1	10	0	Co-exposure to ticks	Passive surveillance
2020	Sanlian Street, Shushan District	5	1	23	0	Human-to-human	Hospital Report
2021	Suwan Town, Chaohu City	2	0	4	0	Co-exposure to ticks	Passive surveillance

### 2.5 Latent infection rate of SFTSV in the population

In 2016, 385 people were surveyed in the four SFTS multi-incidence counties, with a latent infection rate of 6.75%. 403 people were surveyed in 2021, with a latent infection rate of 10.91%, and the difference in the latent infection rate of SFTSV between the two surveys was statistically significant (χ^2^ = 4.453, *P* = 0.033). Among them, the difference in SFTSV latent infection rate between the two surveys in Lujiang County was statistically significant (χ^2^ = 3.867, *P* = 0.049), and the differences in SFTSV latent infection rate in the other three multi-incidence counties were not statistically significant (see Table 3).

**Table 3:** Two survey results of SFTSV latent infection rate in SFTS multi-incidence counties [n/%]

**Year**	**2016**		**2021**		**χ2**	***P***
	
	**Number**	**Infection situation**	**Number**	**Infection situation**		
Chaohu	96	4/4.16%	100	7/7.00%	0.742	0.389
Feixi	96	8/8.33%	101	6/5.94%	0.427	0.514
Feidong	96	6/6.25%	100	13/13.00%	2.781	0.095
Lujiang	97	8/8.24%	102	18/17.64%	3.867	0.049

Total	385	26/6.75%	403	44/10.91%	4.543	0.033

## 3 Discussion

The main findings of this study showed that the incidence of SFTS in Hefei from 2015 to 2021 showed an overall increasing trend, and cluster outbreaks occurred from time to time. The number of affected towns (streets) of SFTS cases in the city increased significantly, and the affected areas continued to expand. In addition, SFTSV has a certain latent infection rate in healthy people in multiple counties, and the prevention and control form of SFTS in Hefei is relatively severe.

In recent years, the overall trend of SFTS incidence in Hefei from 2015 to 2021, although lower in the previous years, is increasing, with an average annual reported incidence rate of 0.65/100,000, which is higher than the average annual incidence rate in Henan Province during 2017–2020 (0.46/100,000) [20], which indicates that the form of SFTS prevention and control in Hefei is more severe. The increased incidence rate of SFTS after 2020 is consistent with the overall increasing trend of the epidemic incidence rate in China [9], which may be related to the increase in the distribution and density of ticks, the increase in the frequency of people’s outdoor activities, the enhancement of residents’ awareness of seeking medical treatment, the improvement of diagnosis awareness and detection ability of medical institutions, and the enhancement of surveillance sensitivity [21]. The average annual case fatality rate was 9.73%, which was lower than the currently reported level of 20% case fatality rate [22], probably due to the lack of disease tracking and reporting of post-discharge SFTS cases, which underestimates the case fatality rate.

SFTS is epidemic mainly in the areas of Anhui, Henan and Hubei near the Dabie Mountains [16], and it can be assumed that the Dabie Mountains have a geographical and ecological environment suitable for the survival of vector ticks and the maintenance of the SFTVS cycle. The top 4 counties in Hefei with the highest incidence rates are located precisely in the remnants of the Dabie Mountains, and the counties are mostly hilly landforms, thus had the characteristics of regional distribution. In contrast, the main urban area of Hefei and Changfeng County, which are located in the plains, also reported SFTS cases probably due to the increase in greening rates in recent years, or citizens or household pets entering green areas and acquiring infection. The results of this study suggest that, similar to other natural epidemic diseases [23], with the rapid acceleration of China’s urbanization process and the increase of urban population density, rural infectious diseases have gradually begun to pose a major threat to urban residents. Therefore, in addition to paying attention to natural factors, we can further explore the internal driving and influencing factors of the expansion and spread of SFTS from social factors, such as gross domestic product (GDP) and high living standard demand.

The combination of natural and social factors led to the coexistence of two patterns of SFTS incidence in Hefei: highly disseminated and locally spatially clustered. Highly disseminated cases are not only reflected in the number of towns (streets) with SFTS cases increasing year by year (*P* < 0.05), but also in the tendency of covering all towns (streets) in the county and spreading to the main urban area; it is also reflected in the majority of towns (streets) with 1–5 cases (12.50%–36.51%). Local spatial cluster suggests the risk of clustered cases.

The prevention and control guidelines published in 2010 stated that there are two types of clustered outbreaks: co-exposed ticks and human-to-human transmission [18], and several SFTS human-to-human clustered outbreaks have been reported in China and Korea [24], and eight clustered outbreaks also occurred in Hefei from 2015–2021, with epidemiological investigations considering five clustered outbreaks of co-exposed ticks and three of human-to-human transmission. Clustered outbreaks of co-exposed ticks can be well explained. Continuing cases of interpersonal transmission were mainly family members caring for patients [11, 25] and medical staff who participate in nursing treatment and nursing [12], and subsequent cases were also mainly exposed to blood or bloody respiratory secretions without secondary protection [26], although inhalation of aerosols produced after vomiting blood from patients cannot be excluded from infection [27]. However, the difference in the case fatality rate between both types of clustered outbreaks was not statistically significant (P = 1.000), while a study by Zhang AP and others [28] showed that the clinical symptoms and prognosis differed between the different routes of infection, with SFTS patients infected by tick bites being sicker and more likely to have a poor prognosis. The difference in the latent infection rate due to the two types of clustered outbreaks was not statistically significant (P = 0.127) and may be related to the higher level of awareness and protection against SFTS in the population exposed to the indicated cases. The difference in the case fatality rate due to the two different discovery pathways was not statistically significant (P = 0.624) and may be related to the fact that most of the renewed cases were in the middle-aged population with a stronger organism resistance to infection.

SFTSV has a certain latent infection rate in healthy population [29]. It was found that the latent infection rate of SFTSV in four multi-prevalent counties in Hefei increased from 6.75% in 2016 to 10.91% in 2021, which was lower than 11.27% in the survey of high prevalence areas in Henan Province [16], but higher than 6.3% in the healthy population in non-endemic areas in China in 2017 [30], which indicated that the latent infection rate in multi-prevalent counties in Hefei in 2021 compared with 2016 all significantly increased, and the latent infection rate of SFTSV has risen to a higher level. This may have the following reasons: with global warming in recent years, the rise of temperature may change the lifestyle of individuals [31], and the frequency of outdoor activities of hosts will increase [32], resulting in greater contact between hosts and ticks; as while as, the increas of vegetation cover for environmental protection is more favorable for tick survival [33], ticks are natural hosts of SFTSV, while domestic or wild animals are acting as amplified hosts, living environment and outdoor labor in living in the middle of the village/community, frequent tea picking, keeping tick-attached poultry livestock, and exposure to wild animals are risk factors [34, 35]. Also, in recent years, in the context of the coronavirus (COVID-19) disease epidemic, more local residents are working at home in agriculture or involved in other outdoor labor, thus increasing the chances of exposure and infection. The current situation of increasing both the latent infection rate and the incidence rate suggests that the level of latent SFTSV infection rate is closely related to the level of SFTS incidence.

The shortcomings of this study are as follows: firstly, SFTS as a non-notifiable infectious disease, which may be under-reported; secondly, the transmission details of human-to-human clustered outbreaks are also inferred on the basis of epidemiological surveys, which is difficult to quantify; third, the sample size of the serological survey was small; fourth, spatial statistical methods such as spatial autocorrelation were not used.

In summary, the incidence rate of SFTS in Hefei is at a high level, and the incidence is obviously regional, with an expanding trend in the extent of the epidemic involved and the existence of highly disseminated and locally clustered incidence patterns, with co-exposure to ticks accounted for the majority of clustered outbreaks and also human-to-human outbreaks. The latent infection rate also rises to a higher level in multi-prevalent counties. Therefore, we should strengthen the disease monitoring of SFTS, including follow-up after discharge, in terms of popularizing public knowledge, information and practice technology. Ongoing dynamic serology surveillance to understand the dynamic pattern of SFTSV infection and, if necessary, symptom surveillance and studies of host and meteorological environmental factors in mildly or asymptomatically infected individuals in the epidemic population to discover the causes of the epidemic and to accurately grasp the citywide epidemiologic characteristics of SFTS.

## Declarations

### Authors’ information

All authors are from Hefei Center for Disease Control and Prevention, Hefei, China.
